# MIGHTY: a virtual solution to global health education

**DOI:** 10.5116/ijme.674f.28ad

**Published:** 2024-12-23

**Authors:** Kristin Maletsky, Kimberley F. Alali, Aya Fanny, Heather L. Crouse, Justin Moher, Morgan Congdon

**Affiliations:** 1 Department of Pediatrics, Perelman School of Medicine at the University of Pennsylvania, Children's Hospital of Philadelphia, Philadelphia, Pennsylvania, USA; 2 Department of Pediatrics, Baylor College of Medicine, Texas Children's Hospital, Houston, Texas, USA; 3 Department of Pediatrics, University of Washington School of Medicine, Seattle Children's Hospital, Seattle, Washington, USA

**Keywords:** Global health, medical education, online learning; COVID-19

## Introduction

The COVID-19 pandemic disrupted global health (GH) education as international electives were canceled for trainees, depriving them of experiential learning instrumental for developing knowledge and skills related to caring for patients in resource-limited settings.^1^^,^^2^  Born from this need, the Association of Pediatric Program Directors’ (APPD) GH Learning Community Steering Committee tasked a group of fellows and faculty with expertise in GH and medical education to identify a solution and reconsider previous practices for GH education.

This article outlines the virtual curriculum design, reviews potential barriers to implementation and uptake, and highlights ways in which global health educators can foster interconnectedness and international community-building.

### Approach

Our intervention aimed to expand knowledge of best practices in pediatric GH for trainees through case-based discussions, which were facilitated by content experts. To do so, we developed a virtual GH series for pediatric trainees (e.g., medical students, residents, and fellows) representing multiple institutions called the Multi-institutional Global Health Trainee Yearlong (MIGHTY) curriculum.

### Our Solution

First, in considering both curricular goals and feasibility, we selected the traditional morning report-style presentation.^3^ We crafted a template for case presentations including a summary of the patient’s presenting history, physical exam, and workup, followed by the differential diagnosis and plan for further evaluation and management, culminating in an in-depth discussion of the final diagnosis. All aspects of the presentation incorporated audience participation. We then determined core clinical topics relevant to pediatric trainees based on a publicly available Foundations in Global Health course outline and lecture series.^4^ Utilizing the APPD GH listserv, we solicited pediatric trainee volunteers with international clinical experience to present cases. Finally, we matched each trainee with a faculty content expert and facilitated the inclusion of insight from international partners through livestream participation or pre-recorded content when they were unable to join in person. Session participants were recruited from the same listserv and by word of mouth as the series grew. This educational initiative was reviewed by the Children’s Hospital of Philadelphia and Baylor College of Medicine Institutional Review Boards and met exemption criteria.

From March 2021 through March 2022 we held a total of eight hour-long livestream sessions. Sessions were advertised through national platforms such as the American Academy of Pediatrics Section on Global Health and the APPD GH Learning Community with typically 75-130 RSVPs and 20-30 attendees. Audience participation was encouraged through both live dialogue and utilization of the chat feature. We also developed a publicly available website to house session recordings and resources for future use (with 1592 view counts to date).^5^

### Evaluation

Our team regularly reviewed attendee feedback collected through a post-session survey and made iterative adjustments to subsequent sessions, including additions to the content and variation in the timing of sessions. We conducted, recorded, and transcribed semi-structured interviews with key stakeholders, including presenters, content experts, and attendees. Interview guides were created with a focus on implementation and uptake, grounded in communities of practice theory. The interviews addressed three domains: 1) perceived benefits of attending the sessions, 2) potential barriers to successful delivery, and 3) opportunities for improvement in future sessions. We utilized both deductive and inductive approaches to the thematic analysis of interview transcripts, identifying codes that we categorized into overarching themes (Table 1).

**Table 1:** Major themes and representative quotations from key stakeholder interviews

Theme	Representative Quotations
Inclusion of international colleague expertise adds richness and expertise	“...when you actually involved somebody who is from and currently working in a low- or middle-income country. Including them adds a kind of reality to the case being able to hear their perspective from being in the field.” (Faculty attendee)
Educational value of case-based global health content	“I feel not only is it relevant to global health and major topics that are affecting other countries, but it's also still relevant for being a resident and learning medicine as a whole.” (Resident attendee)
	“You have selected topics that are very relevant, and I think using a case-based approach and gradually unveiling the diagnosis and guiding the whole session around a particular topic like you did but allowing enough discussion and freedom to explore related issues, has been really successful…the only downside was I felt like we didn't have enough time.” (Faculty expert)
Community-building and networking opportunities for trainees and faculty to learn from colleagues	“…a sense of network building and community building. Getting to meet and even hear names in the field that you may have never heard of before at other institutions who are passionate about global health.” (Fellow presenter)
	“I think you've figured out a really good way to harness talents across multiple institutions...I've been exposed to a lot of people who are working in global health that I didn't know before....it was also just really smart in terms of resources and bringing together different types of experts and cases to use the multi-institutional approach.” (Faculty attendee)

### Implications

The virtual sessions facilitated engagement of a diverse group of trainees and faculty from institutions across the country, together with faculty global partners representing six countries, and successfully increased self-reported knowledge of common topics in pediatric GH based on post-session surveys. Our work builds upon prior studies in nursing, undergraduate, and graduate medical education literature that have outlined examples of reciprocal partnerships between high-income and low-income countries and work that has described challenges to virtual interinstitutional collaborations within GH education.^6^^,^^7^ Additionally, attendees shared that while the sessions offered an accessible forum for disseminating knowledge, they also fostered a sense of community and enabled networking, which were incredible unanticipated benefits during a time when many in the GH field felt disconnected. In the future, virtual GH educational initiatives should intentionally cultivate a sense of community and promote networking opportunities instead of solely focusing on content delivery.

Our endeavor was met with both successes and challenges. One of the most significant contributing factors to the successful implementation of the intervention was the widespread network of trainees our team had access to, especially through the APPD GH Learning Community listserv. This APPD GH network enabled our team to identify trainees with GH experience who had an interest in presenting cases, along with a robust faculty group with content expertise. We also leveraged the global partnerships at our home institutions to identify international experts and enhance both the conversations and the relevance of the selected case topics. Additionally, the use of technology, including the ability to generate livestream video conferences that could be viewed by participants across the globe, was one of the largest contributors to the success of the intervention. Finally, the unanticipated benefit of community-building that occurred during these sessions emerged as a key factor that encouraged participants to join for multiple sessions.

Regarding challenges, one significant obstacle was identifying optimal session timing to maximize attendance given that our audience and international experts spanned several time zones. Responding to this challenge in coordinating with international partners in particular, our team introduced pre-recorded video content to facilitate inclusion of crucial perspectives during sessions when experts were unable to join live due to issues with timing. Given that insight from international participants was invaluable, future endeavors should prioritize inclusion of such content experts. Additionally, given the time-sensitive need for GH education content, the initial sessions were created without a framework in place, which delayed efficient and consistent content development. As such, our team quickly developed guidelines for content development, including expectations for both the trainee and faculty members, and templated slides to facilitate both timely development of presentations and to ensure standardization in the delivered product.

Recognizing the continued educational benefits this series can deliver in the post-pandemic setting, we aim to formalize a partnership with pediatric GH training programs, enabling continued and consistent delivery of content for the series. Logistics as it relates to identifying and assigning speakers, including among international partners, and scheduling sessions remains a key priority for the longevity of this curriculum and its leadership team. We also aim to expand our efforts to include international pediatric trainees, both as case presenters and attendees, which will not only generate richer discussions, but help to broaden connections and enable opportunities for increased networking and partnerships in an ever-growing GH community. In addition to live case conferences that facilitate networking, our curriculum is available through the public webpage, which can be used to supplement both formal and self-driven asynchronous learning endeavors for trainees and practicing clinicians.

### Acknowledgments

We would like to acknowledge the contributions of the trainees, faculty, and international partners, without whom this series would not have been possible. 

### Conflict of Interest

The authors declare that they have no conflicts of interest.
